# The effect of void creation prior to vertebroplasty on intravertebral pressure and cement distribution in cadaveric spines with simulated metastases

**DOI:** 10.1186/s13018-015-0160-5

**Published:** 2015-01-28

**Authors:** Ka Li, Jun Yan, Qiang Yang, Zhenfeng Li, Jianmin Li

**Affiliations:** Department of Orthopedics, Qilu Hospital, Shandong University, Jinan, Shandong People’s Republic of China

**Keywords:** Percutaneous vertebroplasty, Intravertebral pressure, Metastatic tumor, Spine, Cement distribution, Void creation

## Abstract

**Background:**

For osteoporosis or spinal metastases, percutaneous vertebroplasty is effective in pain relief and improvement of mobility. However, the complication rate (cement extravasation and fat embolisms) is relatively higher in the treatment of spinal metastases. The presence of tumor tissue plays a significant role in intravertebral pressure and cement distribution and thereby affects the occurrence of complications. We investigated the effect of void creation prior to vertebroplasty on intravertebral pressure and cement distribution in spinal metastases.

**Methods:**

Eighteen vertebrae (T8–L4) from five cadaveric spines were randomly allocated for two groups (group with and without void) of nine vertebrae each. Defect was created by removing a central core of cancellous bone in the vertebral body and then filling it with 30% or 100% fresh muscle paste by volume to simulate void creation or no void creation, respectively. Then, 20% bone cement by volume of the vertebral body was injected into each specimen through a unipedicular approach at a rate of 3 mL/min. The gender of the donor, vertebral body size, bone density, cement volume, and intravertebral pressure were recorded. Then, computed tomography scans and cross sections were taken to evaluate the cement distribution in vertebral bodies.

**Results:**

No significant difference was found between the two groups in terms of the gender of the donor, vertebral body size, bone density, or bone cement volume. The average maximum intravertebral pressure in the group with void creation was significantly lower than that in the group without void creation (1.20 versus 5.09 kPa, *P* = 0.001). Especially during the filling of void, the difference was more pronounced. Void creation prior to vertebroplasty allowed the bone cement to infiltrate into the lytic defect.

**Conclusions:**

In vertebroplasty for spinal metastases, void creation produced lower intravertebral pressure and facilitated cement filling. To reduce the occurrence of complication, it may be an alternative to eliminate the tumor tissue to create a void prior to cement injection.

## Background

The spine is the most common location for skeletal metastases in patients with cancer [1,2]. Spinal metastases are found in approximately 36% of patients dying from malignant neoplasm [2]. The most frequent sites of primary tumors are the breast, prostate, and lung. Spinal metastases can cause local pain, pathological fracture, and neurologic compromise. Conservative treatments, including bed rest, back bracing, narcotic analgesics, radiotherapy, chemotherapy, and biphosphonates, do not provide stability of the spine. Although conventional surgical interventions can provide pain relief, decompression of neurologic elements, and improvement of biomechanical stability [3], patients with metastases may be unable to safely bear them due to the high morbidity related to surgery. Hence, the treatment of spinal metastases remains largely palliative [4]. Minimally invasive techniques with a lower morbidity than conventional surgical interventions can afford palliation and are gaining increasing popularity.

Percutaneous vertebroplasty (PVP) is a minimally invasive procedure consisting of injecting bone cement into structurally weakened or destructed vertebrae under radiological guidance, and has been successfully applied in the pain management of osteoporotic vertebral compression fractures [5], hemangiomas [6], and myeloma [7,8]. Studies have revealed that PVP is effective for spinal metastases in pain relief and improvement of mobility [9-11]. Although it does not improve the survival outcome, pain relief and improved mobility are reasonable goals for patients with metastases whose life expectancies are short [9]. However, the frequency of complications which include cement extravasation and fat embolisms is 10% in PVP for metastases [12]. Studies have reported that the incidence of cement leakage in PVP for spinal metastases is approximately 50% to 85% [13-15]. A retrospective research even reported that 423 cement leakages occurred in the treatment of 304 metastatic vertebral bodies using PVP [16]. Fortunately, only a small percentage of leakages result in symptomatic complications. Other complications include embolisms due to the bone marrow, tumor fragments, or bone cement. The presence of tumor tissue in the vertebral body contributes to a higher intravertebral pressure and unsatisfactory cement distribution [17]. It is necessary to explore a method to reduce the adverse effects of tumor tissue and investigate the underlying basis of the method in vertebroplasty.

We hypothesize that void creation by removing tumor tissue prior to vertebroplasty may decrease the intravertebral pressure and improve bone cement distribution. The purpose of this study was to research the effect of a void created by tumor elimination prior to vertebroplasty on intravertebral pressure and cement distribution in simulated spinal metastases.

## Methods

Eighteen intact vertebrae (T8–L4) were harvested from five human cadaveric spines and stored at −20°C. The average age of the donors was 71.4 ± 6.1 years (range, 63 to 79 years; three males and two females). The specimens were randomly allocated for two groups of nine vertebrae each. For each vertebra, computed tomography (CT) was taken to rule out pathological lesion or abnormality and to measure the vertebral body size. The vertebral body size was obtained by measuring vertebral body height and calculating cross-sectional areas based on an ellipsoid shape at the superior, middle, and inferior planes. The periosteum and intervertebral discs were left to maintain the integrity of the vertebral body. The specimens were thawed at 3°C overnight and then placed in a saline bath at 37°C for 2 h prior to testing to get close to human body temperature as much as possible. The research was approved by the Medical Ethical Committee of Qilu Hospital, Shandong University.

To create a defect of which the volume represented 15% of the vertebral body volume, a central core of cancellous bone was removed from the middle of the lateral wall of the vertebral body with a cylindrical trephine and a curet, without breaking through the opposite posterior and anterior vertebral wall. The muscle of the thigh, excised from living New Zealand white rabbits, was chopped and ground until it became a paste. Then, the paste was placed in a syringe and injected into the defect. The animal experimental protocol complied with the Animal Management Rules of the Chinese Ministry of Health (Document No. 55, 2001) and was approved by Animal Care and Use Committee of Shandong University. The volume of muscle paste was equal to 100% or 30% volume of defect for each vertebra in the two groups, respectively, to represent no void creation or void creation. Then, the hole in the lateral wall of vertebral body was sealed with polymethylmethacrylate bone cement. The bone density of each vertebra was determined by the dry weight and trabecular size of the removed bone core, a methodology previously described by Reidy et al. [17].

Two 11-gauge bone biopsy needles (SterilabHiggins KB S.r.l, Buccinasco, Italy) were inserted through both pedicles of each vertebra under fluoroscopic control. The tip of one cannula used for cement injection was placed into the defect. Another cannula connected to a digital manometer (8230, AZ Instrument Corp., Taichung, Taiwan) was placed just beyond the pedicle-body junction.

The volume of radio-opaque polymethylmethacrylate bone cement (Simplex P, Stryker Howmedica Osteonics, Mahwah, NJ, USA) injected was 20% of the vertebral body volume. The bone cement that had been cooled to 4°C for 24 h was mixed according to the manufacturer’s instructions (20 mL monomer to 40 g powder). The cement was loaded in a 10-mL injection syringe which was connected to the injection cannula. To keep a constant injection speed (3 mL/min), the injection syringe was placed on a holding jig and the plunger was coaxially attached to an electromechanical universal testing machine (CMT5105, SANS, Shenzhen, China). The room temperature was strictly kept at 22°C, and the time from mixing the bone cement to injection for all specimens was consistent. The intravertebral pressure was recorded by a digital manometer throughout the process of cement delivery.

CT scans were taken to assess bone cement extravasation in specimens after vertebroplasty. Finally, the vertebrae were axially sectioned to visualize cement distribution in the vertebral body.

Data was calculated and presented as mean ± standard deviation for vertebral body size, bone density, cement volume, and intravertebral pressure. Statistical analysis was done using the Mann-Whitney *U* test to compare the difference between the two groups in terms of intravertebral pressure. Fisher’s exact test was used for the analysis of extravasation. A *P* value of ≤0.05 was considered significant for all statistical analyses. Statistical analysis was carried out using SPSS V17.0.

## Results

**Figure 1:**
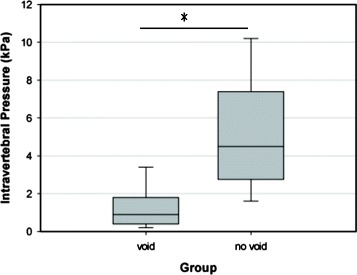
**The comparison of maximum intravertebral pressure during vertebroplasty between two groups.** The average maximum intravertebral pressure was significantly higher in the group without void creation than in the group with void creation (5.09 versus 1.20 kPa). *P < 0.05.

**Figure 2:**
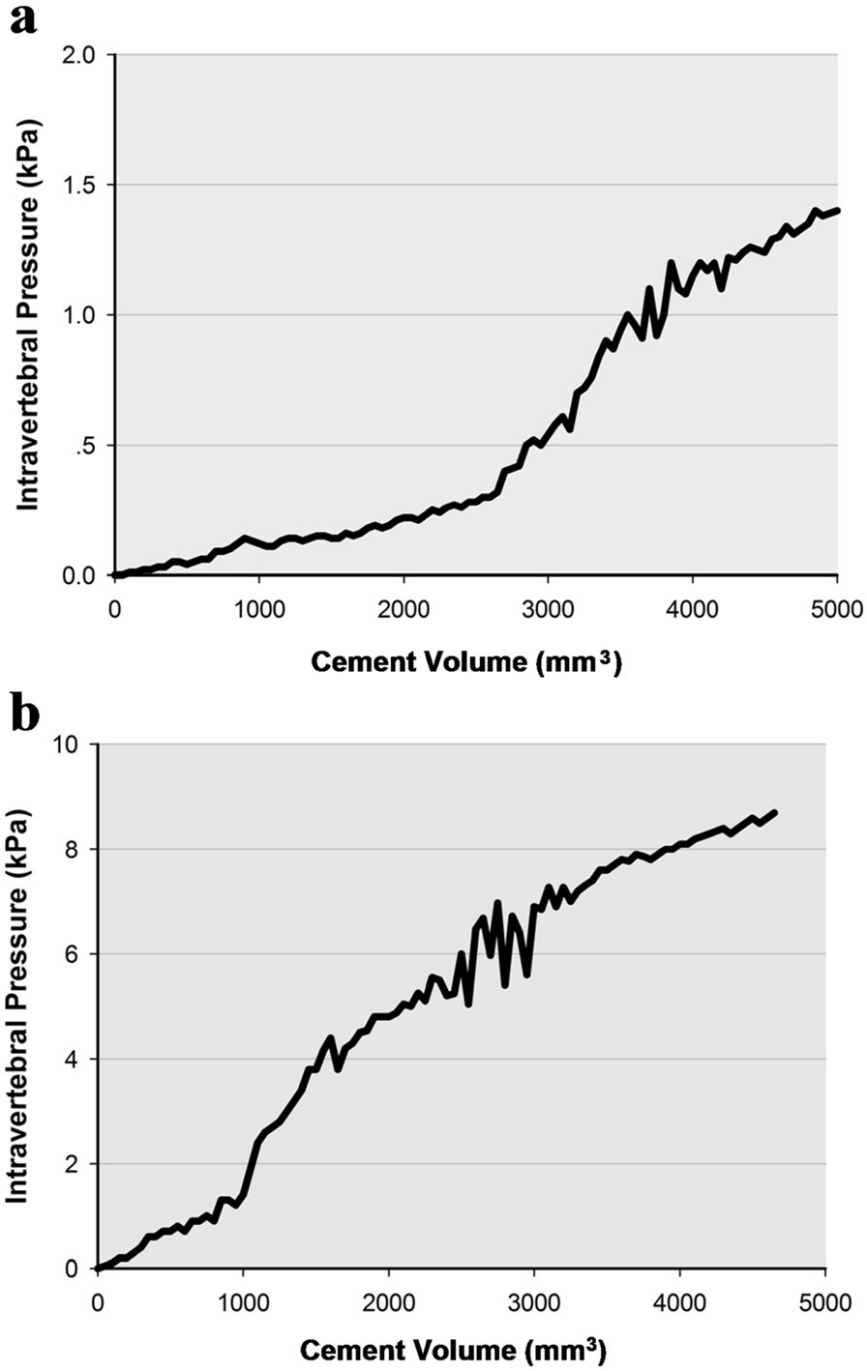
**The pressure-volume curves during cement injection. (a)** In a specimen with a void, the intravertebral pressure remained very low (about 0.3 kPa) until the filling of void is completed. The maximum intravertebral pressure was 1.4 kPa, and the volume of injected cement in this specimen was 4,965 mm^3^. **(b)** In a specimen without a void, the intravertebral pressure rose constantly during cement injection. The maximum intravertebral pressure was 8.7 kPa, and the volume of injected cement in this specimen was 4,611 mm^3^.

**Figure 3:**
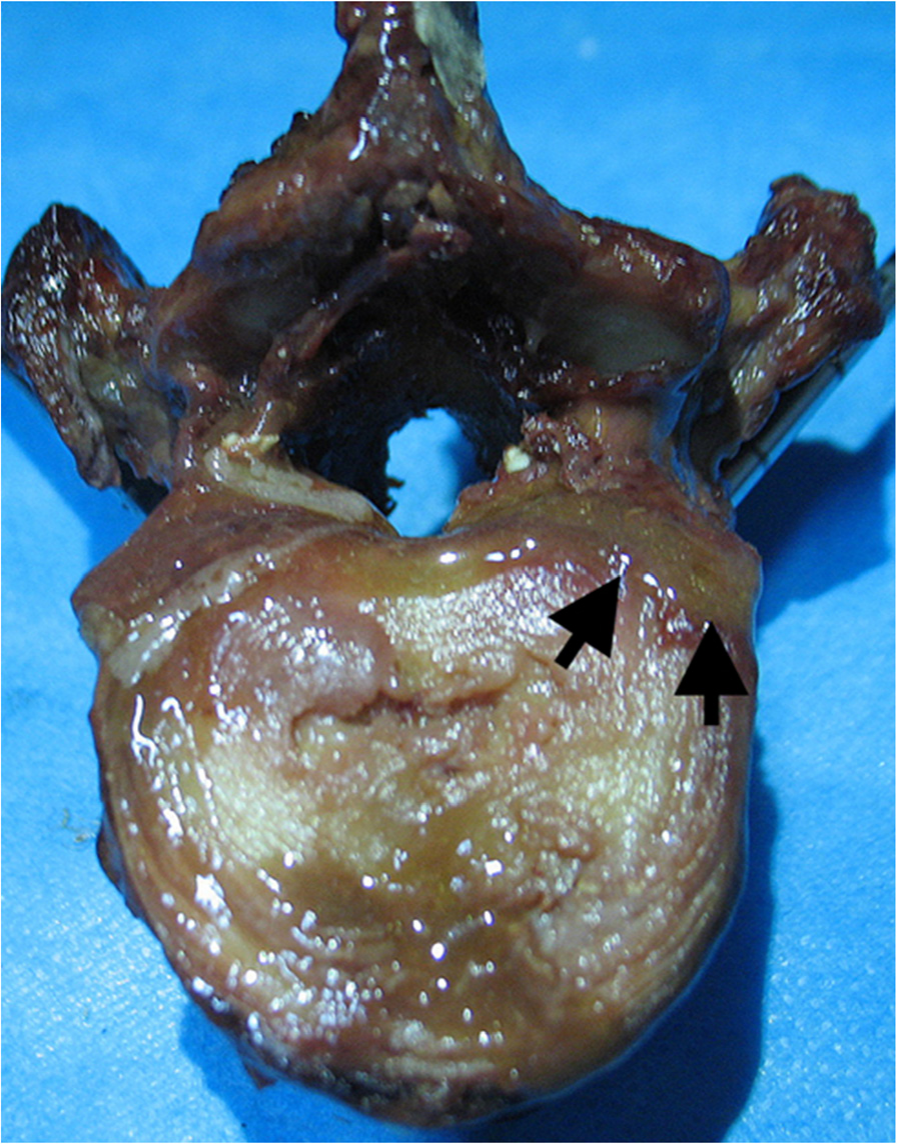
**Photograph of a vertebra without void creation after cement injection.** The bone marrow (*black arrows*) was shown to be displaced out of vertebral body through defects in the cortical shell.

There was no significant difference between the two groups in terms of the gender of the donor, vertebral body volume, bone density, or volume of bone cement (Table 1). The average maximum intravertebral pressure was approximately four times higher in the group without void creation than in the group with void creation (5.09 versus 1.20 kPa). Although there was a lot of variation and overlap, the difference was statistically significant between the two groups (*P* = 0.001, Table 1, Figure 1). Especially during the filling of void, the difference was more apparent. The average intravertebral pressure was 23.25% of the average maximum pressure when the filling of void (10.5% volume of the vertebral body) was just completed in the group with a void. In contrast, 59.72% of the average maximum pressure was reached when 10.5% bone cement by volume of the vertebral body was intruded in the group without a void. While the intravertebral pressure increased over the course of cement injection generally in both groups, the pressure remained very low until the filling of void was completed in specimens with a void. Typical pressure-volume curves in two specimens, which were introduced with a similar cement volume (4,965 and 4,611 mm^3^), from different groups are shown in Figure 2. In addition, bone marrow was released out of the vertebral body through cortical defects in some specimens without void creation during cement injection (Figure 3).

**Table 1:** **Summarized data of the results**

	**Void + VP**	**VP alone**	***P***
Maximum intravertebral pressure (kPa)	1.20 ± 1.04	5.09 ± 2.84	0.001*
Cement extravasation	33.3% (3 of 9)	55.6% (5 of 9)	0.319
Gender of donor (male versus female)	5 versus 4	6 versus 3	1.000
Bone density (g/cm^3^)	0.27 ± 0.074	0.21 ± 0.051	0.085
Vertebral body size (mm^3^)	22,995 ± 6,243	24,222 ± 6,616	0.691
Cement volume (mm^3^)	4,599 ± 416	4,844 ± 441	0.691

*VP* vertebroplasty.

*Significant difference.

**Figure 4:**
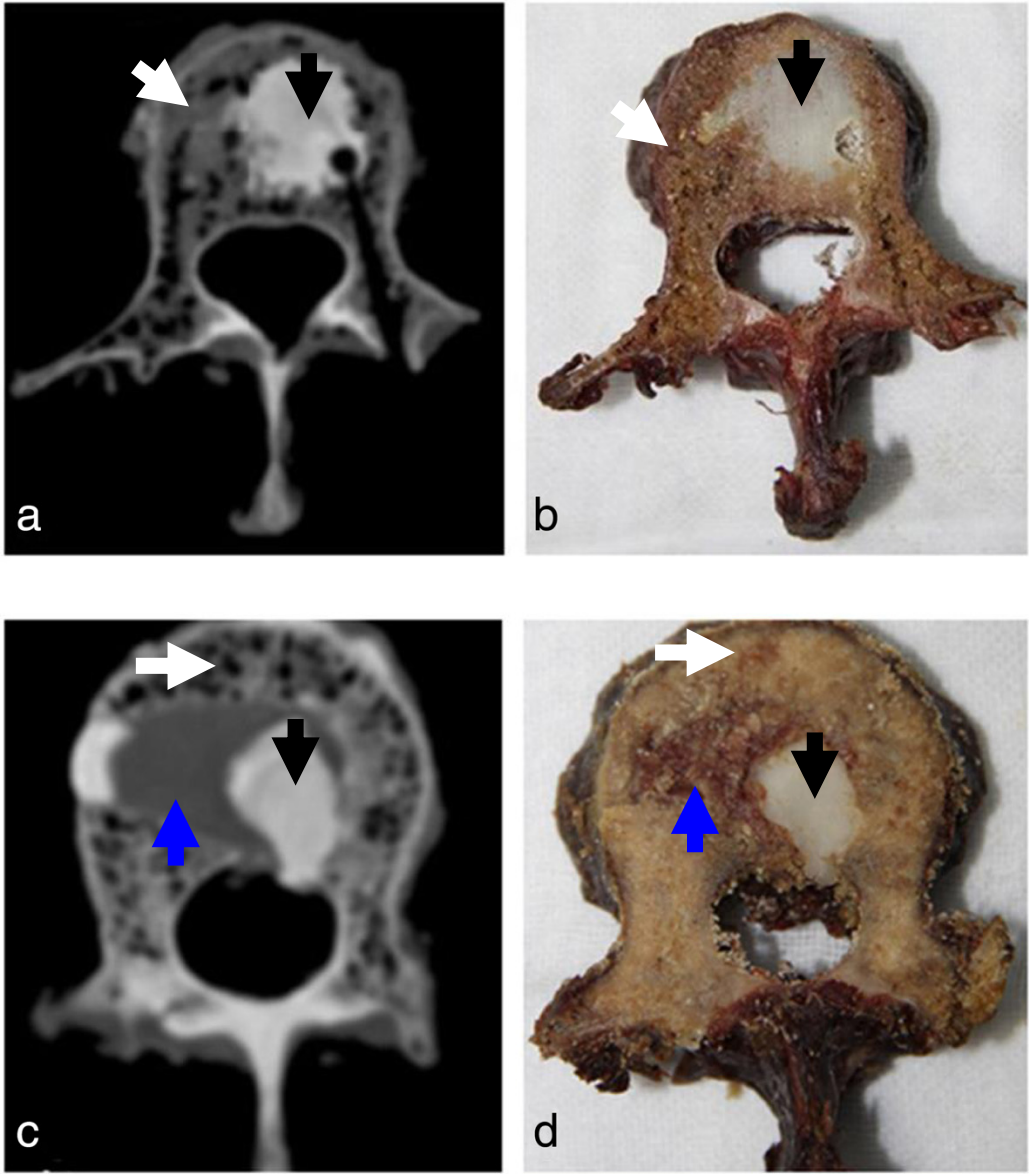
**CT scans and sections showing cement distribution after vertebroplasty. (a, b)** In a specimen with void creation, bone cement was deposited in the cavity and no extravasation occurred. **(c, d)** In a specimen without void creation, bone cement filled the regions lateral and posterior to the muscle paste and extravasated into the spinal canal. *Black arrows*, bone cement; *blue arrows*, muscle paste; *white arrows*, normal bone.

CT scans showed that leakage occurred in five of the specimens without void creation and in three of the specimens with void creation (Table 1). Although the number of extravasation was less in the group with void creation, there was no statistically significant difference between the two groups (*P* = 0.319). Cement extravasation into the spinal canal was present in seven cases. A part of the cement infiltrated into the pedicle with extravasation in one specimen. Following CT scans, the vertebrae were sectioned to assess the bone cement distribution. Bone cement was deposited in the cavity as well as in areas lateral, anterior, or posterior to the muscle paste in specimens with void (Figure 4a, b). However, in specimens without void, the muscle paste remained within the defect and cement filled the regions around the muscle paste, which was accompanied by cement extravasation (Figure 4c, d).

## Discussion

In the current study, we measured and compared intravertebral pressure and cement distribution in vertebroplasty in simulated lytic metastases with and without void creation prior to cement injection.

A previous study has elaborated that metastases led to the decrease of hydraulic permeability of the vertebral body on the account of a relatively higher viscosity of tumor tissue as compared with the bone marrow, which generated higher intravertebral pressure during cement injection [17]. The creation of a void replaces tumor tissue with space and reduces the effect of tumor tissue on the hydraulic permeability of the vertebral body. Consequently, intravertebral pressure decreases with an increase of the hydraulic permeability of the vertebral body. It may explain the decreased intravertebral pressure in the specimens with void creation, especially in the phase of filling of the void, as observed in the present study.

Bone marrow was displaced out of the vertebral body in some specimens without void creation during cement delivery in the study. Animal studies have revealed that fat embolism and hypotension were associated with the elevation in intravertebral pressure, as a result of release of bone marrow contents into the circulation system during vertebroplasty [18,19]. “Snow-flurry,” an indication of small amounts of bone marrow in the bloodstream, was noted by transesophageal echocardiograms in patients when intramedullary pressure was above 6.7 kPa during medullary nailing [20]. In this study, the maximum intravertebral pressure in the specimens with void creation was 3.4 kPa, below the risk level (6.7 kPa) for embolism during medullary nailing [20]. Clinical studies revealed that a vent hole in the distal femur to decrease the intramedullary pressure reduced the occurrence of fat embolism during total hip replacement [21-26]. In addition, an animal study suggested that a vent hole reduced the amount of fat embolism and attenuated the deterioration of mean arterial blood pressure in vertebroplasty [27]. These showed that cardiopulmonary changes were correlated with the elevation of intraosseous pressure and decreased pressure could reduce the risk of cardiopulmonary changes. The current study showed that a void could significantly decrease intravertebral pressure in metastatic vertebrae. Therefore, a void may play the same role, attenuating the severity of cardiopulmonary changes, as vent hole *in vivo*. This should be further verified in prospective clinical studies.

There has been no evidence that intravertebral pressure is a factor influencing the risk of leakage so far. The parameters which had an influence on cement extravasation included the cement viscosity, bone permeability, marrow viscosity, diameter of the extravasation, bone porosity, size of the injection cavity, and bone pore size [28]. In brief, bone extravasation depended on cement viscosity and bone structure. Weisskopf et al. [29] and Reidy et al. [17] revealed that no correlation between intravertebral pressure development and cement extravasation could be established. However, when bone cement augments a specific region in the vertebral body, it is required to extrude the tumor tissue out of the region of metastases. Due to the higher viscosity of tumor tissue, bone cement will be confronted with larger resistance in the region occupied by the tumor and tend to follow a path of least resistance within the vertebral body, resulting in cement extravasation or distribution in the region without tumor. Therefore, we think that void creation decreases the intravertebral pressure, and thereby, bone cement is prone to deposit in the cavity with less cement extravasation, which is demonstrated by the results of this study and not consistent with previous studies. Although less extravasation occurred in the group with void creation compared with the group without void creation (three versus five), no statistically significant difference was found between the two groups. The small sample size is an important reason for the result, and other factors including cement viscosity and individual anatomy of vertebrae also affect cement extravasation [28,30-32]. The effect of void creation on cement extravasation may be weakened by the factors mentioned above. Furthermore, the current study revealed that void creation prior to vertebroplasty allowed cement to fill the structurally weakened lytic defect, which was identical with previous results reported by Ahn et al. in a cadaveric study [33]. In brief, our findings revealed that the presence of a void led to a reduction of intravertebral pressure and thus to less cement extravasation and improved cement distribution in vertebroplasty.

In other *in vitro* researches about vertebroplasty, a specific volume of bone cement was introduced for all specimens [17,33]. It is convenient to manipulate and calculate, but the identical volume of bone cement may affect the measurement of intravertebral pressure and cement distribution for vertebrae with various volumes. Hence, 20% bone cement by volume was injected into each vertebra in the current study, which is a similar approach to that used in clinical practice. It was demonstrated that the vertebral body required at least 20% filling of polymethylmethacrylate bone cement to improve the mechanical integrity [34,35]. In some situations, when a transpedicular approach is used, the device used for tumor elimination cannot completely remove the tumor as a result of geometric factors related to the location of the lesion away from the needle trajectory [33,36]. To better simulate void creation in practice, 30% muscle tissue by volume filled the defect created by removing a core of cancellous bone in this study.

However, there are limitations in this study as follows. The specimen was dissected from cadaveric spines into individual vertebra. Though the periosteum and intervertebral discs were left to maintain the integrity of the vertebral body, the defects in the cortical shell could lead to pressure relief during cement injection, resulting in a lower intravertebral pressure. Tumor tissue simulated with muscle tissue from an animal could not fully represent the characteristics of metastases occurring naturally, such as growth form, heterogeneity, stiffness and permeability, and so on. It may contribute to the inaccuracy of intravertebral pressure measurement. Hence, the *in vitro* model created by individual vertebra from cadaveric spines with muscle tissue could only simulate the spinal metastases to a limited extent. In addition, the small sample size in this study caused an adverse effect on the analysis of cement extravasation data. Due to the *in vitro* study, we could not directly obtain the effect of decreased intravertebral pressure caused by void creation on cardiopulmonary changes. Some studies revealed that pre-vertebroplasty tumor ablation using minimally invasive techniques, such as radiofrequency ablation [37,38], Coblation [36,39,40], was feasible, safe, and effective for metastatic spinal lesions in clinical practice. Hence, a prospective and randomized controlled trial with a large patient number is necessary to validate the preliminary results in this study and to further delineate the clinical benefits of void creation prior to vertebroplasty in spinal metastases.

## Conclusions

The high intravertebral pressure and unsatisfactory cement filling appear in vertebroplasty for spinal metastases. The creation of a void contributes to the decreased intravertebral pressure, which potentially reduces the risk of fat embolism and improves cement distribution in spinal metastases. To reduce the occurrence of complications, it may be an alternative to eliminate the tumor tissue to create a void prior to cement injection.

## Abbreviations

PVP: Percutaneous vertebroplasty
CT: Computed tomography
